# The Ca^2+^-actin-cytoskeleton axis in podocytes is an important, non-immunologic target of immunosuppressive therapy in proteinuric kidney diseases

**DOI:** 10.1007/s00467-025-06670-z

**Published:** 2025-01-25

**Authors:** Agnes Hackl, Lutz T. Weber

**Affiliations:** Department of Pediatrics, University of Cologne, Faculty of Medicine and University Hospital Cologne, Kerpener Street 62, 50937 Cologne, Germany

**Keywords:** Glucocorticoids, Cyclosporine A, Tacrolimus, Mycophenolate mofetil, Rituximab, Non-immunologic effect on podocytes

## Abstract

The integrity of the filtration barrier of the kidney relies on the proper composition of podocyte interdigitating foot processes. Their architecture is supported by a complex actin-cytoskeleton. Following podocyte stress or injury, podocytes encounter structural changes, including rearrangement of the actin network and subsequent effacement of the foot processes. Immunosuppressive drugs, which are currently used as treatment in proteinuric kidney diseases, have been shown to exert not only immune-mediated effects. This review will focus on the direct effects of glucocorticoids, cyclosporine A, tacrolimus, mycophenolate mofetil, and rituximab on podocytes by regulation of Ca^2+^ ion channels and consecutive downstream signaling which prevent cytoskeletal rearrangements and ultimately proteinuria. In addition, the efficacy of these drugs in genetic nephrotic syndrome will be discussed.

**Graphical Abstract:**

**Figure:**
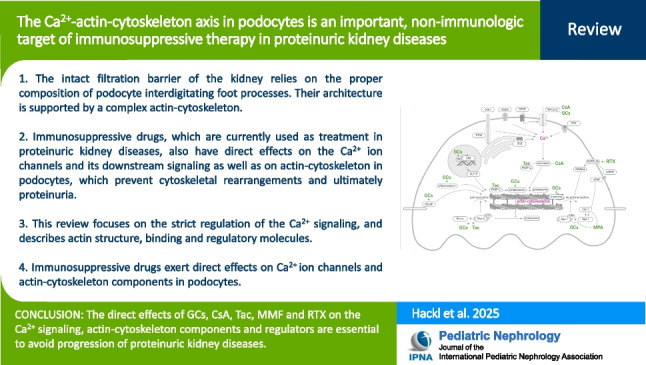
A higher resolution version of the Graphical abstract is available as Supplementary information

**Supplementary Information:**

The online version contains supplementary material available at 10.1007/s00467-025-06670-z.

## Introduction

The filtration barrier of the kidney consists of three layers, the fenestrated endothelium lined with glycocalyx, the glomerular basement membrane (GBM), and the podocytes with the slit diaphragm (SD) bridging their interdigitating foot processes (FPs). The architecture of podocytes is mainly supported by a complex network of the actin-cytoskeleton. Upon podocyte stress or injury, the actin-cytoskeleton of podocytes undergoes rearrangements of the actin network resulting in subsequent FP effacement (FPE) [1]. It has been proposed that FPE initially might be a compensatory attempt of podocytes to prevent detachment. However, if the insult is prolonged, detachment often cannot be avoided. Recent studies have shown that the nephroprotective effect of renin‐angiotensin‐aldosterone system inhibitors are associated with the inhibition of a Ca^2+^ ion channel and its downstream signaling, which prevented cytoskeletal rearrangements in podocytes and proteinuria [2–6]. Other immunosuppressive drugs, which are regularly used to treat proteinuric kidney diseases, have also been shown to exhibit additional direct effects on the Ca^2+^ ion channels and the actin-cytoskeleton beyond their role in systemic immunosuppression and thus directly protect podocytes. Therefore, this review will focus on these drugs and their effect on the Ca^2+^-actin-cytoskeleton axis.

## Ca^2+^ signaling and the actin-cytoskeleton in podocytes

### Ca^2+^ signaling in podocytes

Ca^2+^ signaling is initiated by an increase of the intracellular calcium concentration ([Ca^2+^]_i_). The influx of Ca^2+^ can originate from the extracellular space or intracellular Ca^2+^ stores such as the endoplasmic reticulum (ER) and the mitochondria [7]. The binding of Ca^2+^ induces structural and conformational changes in various calcium-binding proteins, thereby modulating their activity and function in downstream signaling cascades to regulate cell motility and survival. The level of [Ca^2+^]_i_ is meticulously regulated by the coordinated activity of ion channels, molecular pumps and cytosolic Ca^2+^ buffers, all of which cooperate to maintain low [Ca^2+^]_i_ at baseline and enable responsive calcium-dependent signaling pathways.

Ca^2+^ signaling has emerged as a central element in podocyte damage. Kerjaschki suggested that an increase in [Ca^2+^]_i_ is an early event in podocyte injury [8]. Indeed, altered Ca^2+^-signaling has been reported in several circumstances of podocyte injury. Either as a direct cause of focal segmental glomerulosclerosis (FSGS) caused by the gain-of-function mutation in transient receptor potential cation channel member 6 (*TRPC6*) [9] or as a uniform response to stress in this cell type, e.g., in protamine sulfate nephropathy [10] or in complement C_5b-9_ complex-mediated podocyte injury [11]. Moreover, significantly elevated Ca^2+^ levels can be measured in response to podocyte injury using in vivo [Ca^2+^]_i_ imaging [12]. This increase in [Ca^2+^]_i_ leads to cytoskeletal disorganization and FPE [13] and subsequently increases podocyte cell motility in different disease models of podocyte injury in vivo [14].

Due to disease causing human mutations, TRPC6 is the most extensively studied channel in the context of Ca^2+^ signaling in podocytes [15–19]. TRPC6 is located in the podocyte foot processes at or near the site of the SD and contributes to the proposed mechanosensing function of the SD, whereby TRPC6 tightly regulates Ca^2+^ currents and cytoskeletal rearrangement of podocytes [20]. TRPC6 can be activated by an increase in [Ca^2+^]_i_ from the extracellular space (receptor-operated Ca^2+^ entry, ROCE) or from intracellular stores (store-operated Ca^2+^ entry, SOCE) [21–23]. During ROCE, phospholipase C [24] is activated by a G-protein-coupled receptor [25, 26]. The subsequently released diacyl-glycerol directly activates TRPC6 channels to trigger downstream calcium signaling [24, 25]. During SOCE, Ca^2+^ levels in the ER are depleted, which result in the activation of the ER-resident stromal interaction protein 1 [27]. This stimulates calcium-release-activated calcium channel protein 1 (Orai1), allowing Ca^2+^ influx into the cell, which directly activates TRPC channels and promotes the trafficking and the insertion of TRPC channels into the plasma membrane. Importantly, additional evidence supports and highlights the importance of TRPC channels as a critical regulator of calcium signaling in podocytes: ANG II activates ROCE to induce TRPC6-mediated Ca^2+^ influx in podocytes under hyperglycemic condition modelling diabetes [28], ANG II-induced albuminuria is reduced in TRPC6 knockout mice [29] and the inhibition of ANGII by losartan blocks calcium signaling in podocytes [6]. However, the strongest evidence for the pathophysiological relevance of TRPC6 remains the development of FSGS and consequent kidney failure in human patients with a gain of function mutation [9, 30].

TRPC5, calcium-activated potassium channels (BKs; KCa1.1) and store-operated channels (SOCs) have also been implicated in glomerular disease development [31–33]. Furthermore, the ionotropic N-methyl-D-aspartate receptor and purinergic P2X receptors are ligand-gated ion channels that are also thought to play a role in the pathogenesis of glomerular disease [34, 35]. Additionally, the IP_3_ receptor also regulates glomerular shape and podocyte foot process formation through SOCE [36]. Further details can be found in the informative review by Tu et al*.* [37]. Taken together, these results demonstrate the importance of calcium and related signaling pathways in the structure and function of the glomerular filtration barrier.

### Actin-cytoskeleton in podocytes

Podocytopathies are characterized by an altered cytoskeletal architecture (imbalance between polymerization and depolymerization) in the actin-rich FPs of podocytes. They can alter the permeability of the filtration barrier by changing the FP morphology [38]. Signal transduction pathways at the FP that influence cytoskeletal dynamics are controlled by the Rho family of small GTPases and their regulators: Rac1 promotes cell motility, RhoA stimulates the formation of contractile actin cables in vivo or stress fibers in vitro, while Cdc42 initiates actin branching [39–42]. Several studies have closely linked the development of proteinuria with the dysregulation of actin organization in podocytes [43–45]. Mutations affecting actin-related structure, anchors, and regulator proteins in podocytes result in actin-cytoskeleton rearrangement, disrupt the filtration barrier, and subsequently lead to kidney disease [46–50]. In this context, the actin-associated regulatory protein, synaptopodin, has gained special attention: It binds directly to RhoA and thereby prevents the targeting of RhoA for proteasomal degradation and thereby induces stress fiber formation in podocytes [51–53]. Furthermore, nephrin a major component of the SD is linked to the actin cytoskeleton and regulates it *via* the nck and CD2AP adaptor proteins [54, 55]. Mutations in nephrin disturb the actin cytoskeleton [56]. Finally, myosin 1e may be important for the function of actin cables, as mutations in myosin1e are also associated with FSGS [57, 58]. Further details can be found in an excellent review by Blaine et al*.* [59]. Altogether, this data underlines that the proper organization and the dynamic regulation of the podocyte cytoskeleton are vital to the kidney filtration function.

### Regulation of the actin-cytoskeleton via intracellular Ca^2+^ signaling

Ca^2+^ binding can trigger podocyte pathology by activating multiple downstream signal transducers such as calcineurin, calmodulin, and small GTPases. Signaling through the Ca^2+^-activated serine/threonine phosphatase, calcineurin**,** has recently emerged as an important modulator of podocyte function. Calcineurin is widely distributed across many cell types including podocytes. Its most renowned function is the activation of the nuclear factor of activated T-cells (NFAT) [60–63], which upregulates interleukin-2 and induces T-cell response [64]. Additionally, calcineurin renders the actin stabilizer synaptopodin by dephosphorylation accessible to cathepsin L-mediated degradation. This leads to modulation in Rho GTPases activity, cytoskeletal rearrangement and proteinuria [53, 65–70]. Ang II has also been shown to activate calcineurin in a TRPC5-dependent manner [69]. Taken together, calcineurin seems to be a key transducer of Ca^2+^-activated signaling in podocyte injury.

Another important calcium sensor is calmodulin. It is a critical upstream regulator of the Ca^2+^/calmodulin–dependent kinase (CaMK4), which activates Rac1 and suppresses synaptopodin and nephrin leading to the remodeling of the actin-cytoskeleton and a motile podocyte phenotype [71]. Podocyte-specific inhibition of CaMK4 restores synaptopodin expression and protects the actin-cytoskeleton from damage. In addition, the interaction between calmodulin and *MYO9A* is crucial to crosslink actin and to regulate RhoA activity [72]. Importantly, heterozygous loss-of-function mutations in *MYO9A* directly impairing the interaction with actin and calmodulin cause a form of human autosomal dominant FSGS. Finally, calmodulin is also involved in the Ca^2+^-dependent inactivation of TRPC6 channels, which serves as a negative feedback regulation to prevent excess influx of Ca^2+^ [73]. Thus, the disruption of the calmodulin-bridge with TRPC6 can lead to sustained Ca^2+^ elevation, stimulation of downstream signaling cascades and filamentous actin (F-actin) rearrangements. Altogether, calmodulin plays an important role in the fine adjustment of [Ca^2+^]_i_ in podocytes.

It is well known that the activation and the inactivation of small GTPases are mediated by guanine nucleotide exchange factors (GEF)s, which stimulate the exchange of bound GDP by free GTP, and by GTPase-activating proteins (GAP)s, which trigger the hydrolysis of GTP to GDP [39]. Interestingly, the highly [Ca^2+^]_i_ dependent Rho GEF, Arhgef1, has been shown to influence vascular tone and blood pressure in vascular smooth muscle cells in vivo [74]. Moreover, Arhgap24/ FiLGAP inactivates Rac1 [75] and a mutant form of Arhgap24/FiLGAP has been associated with a familial form of FSGS [76]. Further details of the regulation of the small GTPases in podocytes can be found in an excellent review by Saleem et al*. *[77]. Altogether, increased [Ca^2+^]_i_ levels are associated with small GTPase-induced cytoskeletal rearrangements, which in turn alter podocyte motility.

In summary, the downstream signaling of Ca^2+^ converges on the actin-cytoskeleton, and Ca^2+^ is a critical regulator to mediate the dynamic remodeling of the actin-cytoskeleton and to contribute to the regulation of the podocyte motility (Table 1).

**Table 1:** Comparison of the specific alterations in various pathways/targets in relation to the effect on podocytes (injury or protection)

Signal pathway/protein	*Function in podocyte protection*	*Function in podocyte injury*
TRPC6	Balance between TRPC5 and TRPC6	Overactivation e.g. through gain-of-function mutation
Calcium-activated potassium channels (KCa1.1)		Activation enhances Ca^2+^ influx through TRPC6 activation
Store-operated channels (SOCs)		Activation triggers actin remodeling
Ionotropic NMDA receptor and purinergic P2X receptor		Activation enhances Ca^2+^ influx
Calcineurin		Facilitates cathepsin L-mediated degradation of synaptopodin
Calmodulin	Ca^2+^-dependent inactivation of the TRPC6 channel	Through Ca^2+^ /calmodulin–dependent kinase (CaMK4) activation of Rac1 and suppression of synaptopodin and nephrin
Synaptopodin	Prevention of synaptopodin from degradation	Cathepsin L-mediated degradation of synaptopodin through calcineurin
Small GTPases	Activation of RhoA promotes stable actin cables; balance between RhoA and Rac1 activation	Overactivation of Rac1 promotes cell motility
Arhgap24/ FiLGAP	Inactivating Rac1	Mutant form of Arhgap24/FiLGAP is associated with a familial form of FSGS
Nephrin	Major component of the SD, is linked to the actin cytoskeleton and regulates it via adaptor proteins	Mutations in nephrin affect the actin cytoskeleton
Myosin1e		Pathological variants in myosin1e are associated also with FSGS

## The effect of immunosuppressive agents on the Ca^2+^-actin-cytoskeleton

**Fig. 1:**
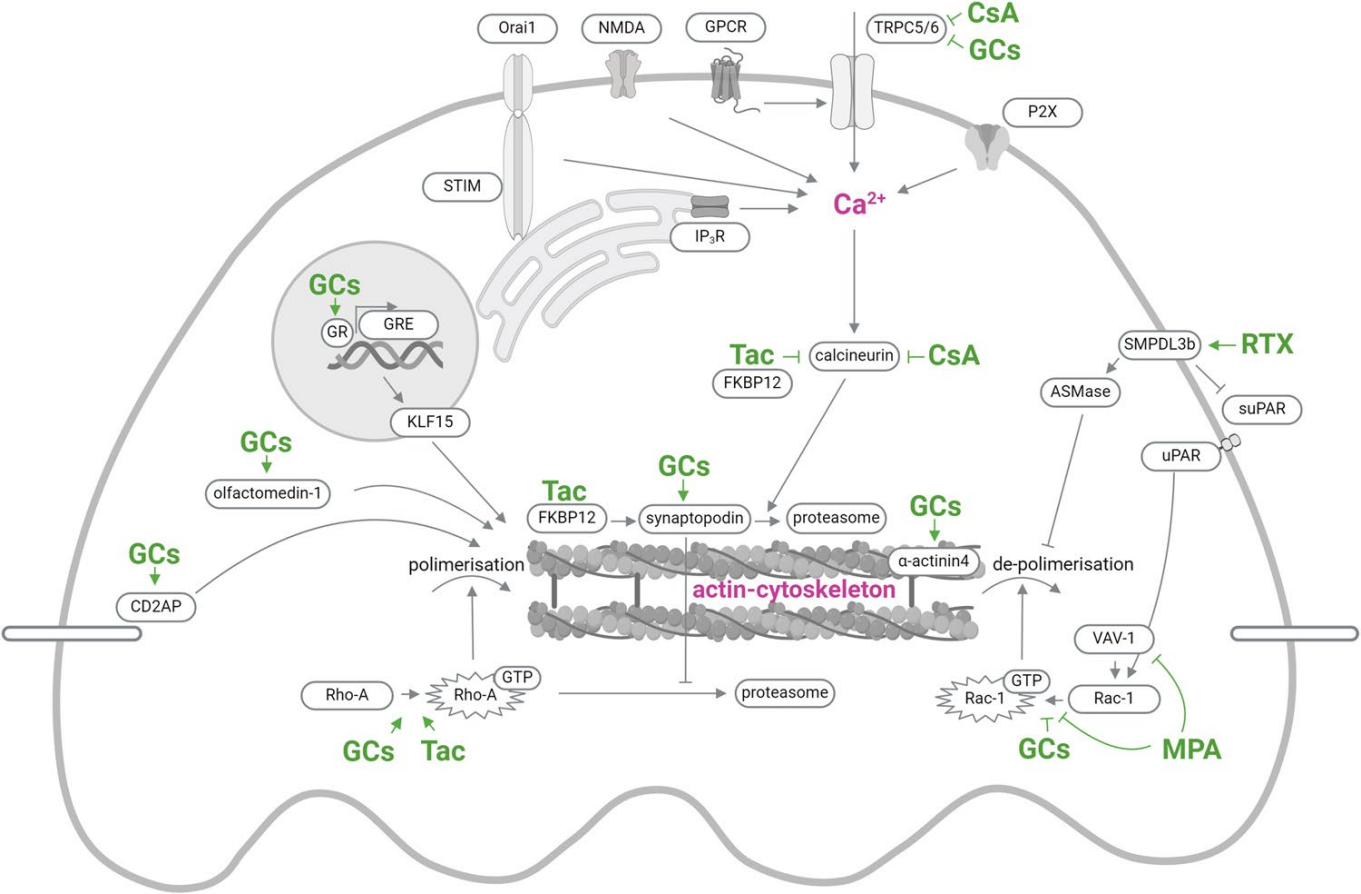
Direct effects of GCs, CsA, Tac, MMF, and RTX on ion channels, actin-cytoskeleton components and regulators in podocytes. Created in BioRender.com. *CsA* cyclosporine A, *GCs* glucocorticoids, *RTX* rituximab, *MPA* mycophenolic acid, *Tac* tacrolimus

Immunosuppressive agents are widely used in the therapy of proteinuric kidney diseases due to their immunotherapeutic or anti-inflammatory therapeutic effects [78]. However, there is growing evidence that these agents may additionally directly target podocytes via the Ca^2+^-actin-cytoskeleton axis and enhance stability of actin filaments (Fig. 1; Table 2).

**Table 2:** Summary of the specific molecular mechanisms of podocyte protection induced by each of the various drugs

*Immunosuppressive agents*	*Effect on podocytes*
***Glucocorticoids***	Blocking TRPC6 signaling
	Activation of the nephrin gene promoter and support of nephrin`s phosphorylation
	Upregulation of CD2AP adaptor protein
	Stabilizing the expression of actin crosslinking protein, α-actinin-4
	Prevention of synaptopodin’s degradation
	Diminishing Rac1 overactivity and enhancing RhoA activity
	Promoting Krüppel-like factor 15 gene expression
	Inducing olfactomedin-1 expression
***Cyclosporine A***	Preventing synaptopodin's dephosphorylation and degradation
	Decreasing TRPC6 gene expression
***Tacrolimus***	Preventing the degradation of synaptopodin
	Increasing the activation of RhoA
***Mycophenolate-mofetil***	Reducing podocyte’s [Ca^2+^]_i_
	Stabilizing stress fiber formation
	Reducing the expression levels of Vav1 and Rac1 activity
***Rituximab***	Preventing acid-sphingomyelinase down-regulation
	Upregulation of ASMase expression and activity
	Binding soluble urokinase-type plasminogen activator

### Glucocorticoids

For decades, glucocorticoids (GCs) have remained the primary treatment for many glomerular diseases [79]. GCs act predominantly by altering gene expression, but also have secondary non-genomic effects [79]. After binding to a cytoplasmic glucocorticoid receptor (GR), which is expressed in every cell, the complex undergoes nuclear translation to induce transcriptional responses via binding to glucocorticoid response elements. In contrast, non-genomic effects are mediated by binding to GRs located in the cytosol or by direct interaction with the cell membrane [80]. Podocytes have also been shown to express functional glucocorticoid receptors [81–83], and several studies have shown that GCs can change gene expression in podocytes in vitro, demonstrating that podocytes are responsive to GCs, as has been comprehensively summarized by *Broek et al.* [84].

**Fig. 2:**
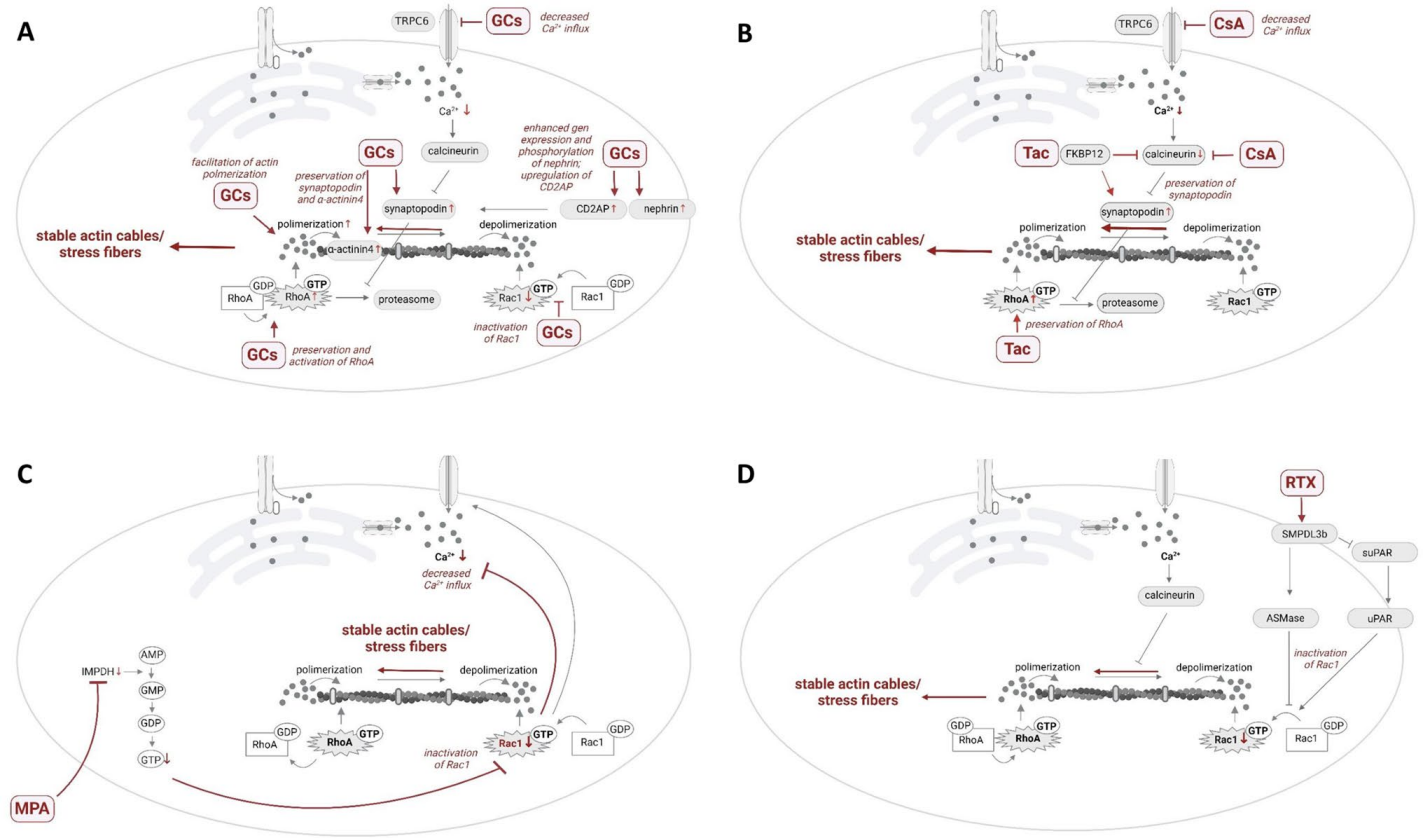
Direct effects of GCs, CsA, Tac, MMF, and RTX on ion channels, actin-cytoskeleton components, and regulators in podocytes. Panels focus on the relevant mechanisms of action of each of the drugs: GCs (**A**), CsA (**B**), Tac (**B**), MMF (**C**), and RTX (**D**). Created in BioRender.com. *CsA* cyclosporine A, *GCs* glucocorticoids, *RTX* rituximab, *MPA* mycophenolic acid, *Tac* tacrolimus

It is well established that GCs can, on one hand, exert significant anti-proteinuric effects by influencing Ca^2+^ signaling: They preserve the structural and functional integrity of the SD by binding and blocking TRPC6 channels [85]. On the other hand, GCs increase actin polymerization and the stability of actin filaments [86, 87]. For example, actin structure and binding molecules have been identified as targets of GCs: They increase gene expression and phosphorylation of nephrin [88, 89], upregulate CD2-associated protein [90], induce olfactomedin-1 expression [91] and promote Krüppel-like factor 15 gene expression, which stabilize the actin cytoskeleton under stress and podocyte injury [92–94]. Furthermore, GCs protect podocytes by stabilizing the expression of α-actinin-4, an actin crosslinking protein that coordinates cytoskeletal organization [95]. Actin regulators are an additional important target group: GC treatment after exposure to proteinuria-inducing agents reduced Rac1 over-activity and podocyte motility [96, 97], increased the stability of actin filaments by stabile synaptopodin expression, and increased RhoA activity [98, 99]. The effects of GCs-induced changes on the actin-cytoskeleton lead to its increased stability and enhanced protection of podocytes against damage (Fig. 2A).

### Cyclosporine A

Cyclosporine A (CsA) is a calcineurin inhibitor that blocks the activation of NFAT in T cells preventing the transcription of cytokines such as Il-2 and Il-4. Recent studies demonstrated that its therapeutic effects extend beyond immune cells. Calcineurin dephosphorylates the actin-associated protein and Rho-GTPase-regulator synaptopodin in podocytes and increases its susceptibility to cathepsin L-mediated degradation. CsA prevents the dephosphorylation of synaptopodin by calcineurin, thereby maintaining the phosphorylation-dependent interaction of synaptopodin with −14–3-3 beta [65] and preventing the degradation of synaptopodin. Therefore, CsA stabilizes the stress fiber formation making podocytes more resistant to the development of proteinuria. Similarly, inhibition of Ca^2+^ channels by CsA as well as the cathepsin L inhibitor reduced FPE, which was induced by the treatment of isolated glomeruli with protamine-sulfate. This suggests that Ca^2+^ signaling through calcineurin- and cathepsin L-dependent cleavage of synaptopodin is essential in the initial stages of glomerular injury and can serve as a podocyte-specific therapeutic target [100]. Accordingly, pre-incubation of mouse podocytes with CsA during treatment with puromycin aminonucleoside increased the expression of synaptopodin and restored the organization of the actin-cytoskeleton [101]. Additionally, CsA decreases TRPC6 expression in doxorubicin nephropathy [102]. These findings (Fig. 2B) clearly demonstrate that the treatment response of CsA in proteinuric kidney diseases extends beyond its immunosuppressive effect and reveals the podocyte as the therapeutic target of choice for glomerular diseases [103].

### Tacrolimus

Tacrolimus (Tac) is another calcineurin inhibitor, that exerts the same effect on calcineurin and downstream inhibition of NFAT activation in T-cells though it lacks clear structural similarity to CsA [61]. Its activity is mediated through the FK506 binding protein 1A (FKBP12). Importantly, FKBP12 is localized to the actin-cytoskeleton and associates with F-actin [104]. FKBP12 knockdown in podocytes leads to alterations in the structure of F-actin, highlighting the importance of FKBP12 expression and function in maintaining the integrity of the actin cytoskeleton [104]. Furthermore, Tac could restore the expression of FKBP12 and enhance the interactions between FKBP12 and synaptopodin to ameliorate FPE in injured podocytes [104]. The stabilizing effect of Tac on the synaptopodin expression is strongly supported by in vitro and in vivo studies [101, 105]. Additionally, Wu et al*.* investigated the calcineurin-related Ca^2+^-actin-cytoskeleton axis and found that it was highly up-regulated in a model of depleted miR-30 and impressively blocked by Tac [106]. Tac also activates the actin-regulator RhoA [107]. Altogether, Tac seems to be able to stabilize stress fibers in podocytes mostly in a Ca^2+^-calcineurin dependent manner, but also directly via actin-cytoskeleton (Fig. 2B).

### Mycophenolate mofetil

The prodrug mycophenolate mofetil (MMF) is activated by esterases in the gut and blood to release the pharmacologically active drug moiety, mycophenolic acid (MPA), which functions as a selective non-competitive inhibitor of inosine 5′-monophosphate dehydrogenase. This is the rate-limiting enzyme in the de novo purine synthesis pathway and thereby impairs lymphocyte proliferation [108]. Importantly, direct effects on non-immune cells, including glomerular cells such as mesangial cells and podocytes, have been attributed to MMF [109]. In a lupus model, MMF reduced expression levels of Vav1 and Rac1 activity, which ameliorated stress fiber formation in podocytes [107]. This finding is further supported by our in vitro experiments, which were designed to exclude the effect of immune cells and study MPA’s direct effect on podocytes: We detected a significant change in 350 genes within 24 h of MPA treatment [110], and these genes were partially related to Ca^2+^-signaling and actin-cytoskeleton regulation. As validation, we showed that MPA treatment was able to completely block the increase in [Ca^2+^]_i_ induced by bovine serum albumin and subsequently stabilize the stress fiber formation. Additionally, we demonstrated in a nephrotoxic serum nephritis model in vivo an improvement of proteinuria treatment and identified a significant reduction in podocyte’s [Ca^2+^]_i_ after MMF treatment as possible underlying mechanisms. This change resulted in a tendency towards structural stabilization of podocyte foot processes [111]. Together, these data suggest a relevant effect of MPA to directly stabilize stress fibers in podocytes (Fig. 2C).

### Rituximab

Rituximab (RTX) is a B-cell depleting chimeric monoclonal IgG1 antibody targeting the CD20 receptor. B-cell destruction is mediated by the Fcγ immunoglobulin receptor, complement activation and trigger of apoptosis [112]. RTX has been shown to be effective in podocytopathies by the depletion of B-cells [113]. RTX also possesses other B-cell-independent effects: By targeting sphingomyelin phosphodiesterase acid-like 3b (SMPDL-3b) in podocyte lipid rafts, RTX regulates acid-sphingomyelinase activity and stabilizes the podocyte actin-cytoskeleton [114]. The inhibition of stress fibers formation in podocytes, following incubation with sera of patients with recurrent FSGS after transplantation, was blocked by treatment with RTX. This response and podocyte viability were dependent on SMPDL-3b expression in vitro, as the knockdown of SMPDL-3b in podocytes abrogated these effects. Furthermore, SMPDL-3b has been shown to increase the stability of the cytoskeleton by binding soluble urokinase-type plasminogen activator receptor with subsequent Rac1 inhibition [115]. No data are currently available on the direct effects of the humanized, and therefore less immunogenic, anti-CD20 antibodies, ofatumumab and obinutuzumab, or the anti-CD38 antibody daratumumab [116]. Altogether, RTX seems to possess a relevant direct effect on podocytes. However, few weeks after administration, RTX is likely no longer present in the circulation, making the duration and relevance of its direct effect on podocytes potentially less pronounced compared to other immunosuppressive agents (Fig. 2D).

### Cyclophosphamide

To date, no data are available about a direct beneficial effect of cyclophosphamide on podocytes. Children with genetic proteinuric diseases also do not respond to cyclophosphamide therapy [117].

## Immunosuppressive therapy in genetic nephrotic syndrome

The non-immunologic effects of the immunosuppressive drugs discussed above raise the question, whether patients with genetic steroid resistant nephrotic syndrome (SRNS) could benefit from an immunosuppressive therapy or not. In terms of CsA, there are some multicenter studies offering sparse data. In a large retrospective multicenter study of CsA treatment, none of the patients with genetic nephrotic syndrome experienced a complete remission and only two (17%) achieved a partial response [66]. In another multicenter study, 3% of patients with genetic SRNS experienced a complete remission, and 16% of patients with genetic SRNS showed a partial remission after CsA therapy [118]*.* The PodoNet Consortium has obtained similar results: Of 74 patients with proven genetic mutations, two patients had complete and 12 partial remissions. Thus, in this series, a total of 14/74 (19%) patients with genetic SRNS had a limited response to immunosuppression with CsA and prednisolone pulses [117]*.* Based on some case reports, children with *WT-1* mutation might constitute a particular group, which could show favorable response to an intensified therapy with CsA and GCs [119, 120]. Interestingly, the authors discuss the potential role of the direct effect on podocytes. In summary, most patients with genetic nephrotic syndrome do not benefit from an immunosuppressive therapy and show significantly lower response rates compared with non-genetic patients. This could be due to a direct effect stabilizing actin-binding proteins, which is missing if these proteins themselves are mutated, leading to drug-resistant damage of the actin-cytoskeleton. As genetic podocytopathies are caused by mutations in multiple distinct proteins, it is not unexpected that their response to therapies differs. The extent of nephrotoxic effect (in case of CsA and Tac) and the subsequent reduction of plasma flow is also difficult to predict. Therefore, it is generally not recommended to administer immunosuppressive drugs in genetic forms of NS. However, in the future, it could be part of an individualized therapeutic concept for patients with genetic NS.

## Conclusion

Immunosuppressive drugs commonly used to treat proteinuric kidney diseases exert direct effects on ion channels, actin-cytoskeleton components, and regulators (Fig. 1) in podocytes. This explains, in conjunction with their effects on immune cells, their clinical effectiveness. In the adaptation to damage, a strict regulation of the Ca^2+^-actin-cytoskeleton axis is crucial for the survival of podocytes. Therefore, utilizing the demonstrated direct effects of GCs, CsA, Tac, MMF, and RTX is an essential part in choosing an individual therapeutic approach. The question, to what extent the non-immune, direct drug actions contribute to sustained effects of immunosuppression, will require continued studies in the future.

## Supplementary Information

Below is the link to the electronic supplementary material.

Graphical abstract (PPTX 355 KB)

## References

[1] Kriz W, Shirato I, Nagata M, LeHir M, Lemley KV (2013) The podocyte’s response to stress: the enigma of foot process effacement. Am J Physiol Ren Physiol 304:333–347. 10.1152/ajprenal.00478.2012 10.1152/ajprenal.00478.2012 23235479

[2] Nitschke R, Henger A, Ricken S, Gloy J, Müller V, Greger R, Pavenstädt H (2000) Angiotensin II increases the intracellular calcium activity in podocytes of the intact glomerulus. Kidney Int 57:41–49. 10.1046/j.1523-1755.2000.00810.x 10620186 10.1046/j.1523-1755.2000.00810.x

[3] Ilatovskaya DV, Palygin O, Chubinskiy-Nadezhdin V, Negulyaev YA, Ma R, Birnbaumer L, Staruschenko A (2014) Angiotensin II has acute effects on TRPC6 channels in podocytes of freshly isolated glomeruli. Kidney Int 86:506–514. 10.1038/ki.2014.71 24646854 10.1038/ki.2014.71 PMC4149864

[4] Hoffmann S, Podlich D, Hähnel B, Kriz W, Gretz N (2004) Angiotensin II type 1 receptor overexpression in podocytes induces glomerulosclerosis in transgenic rats. J Am Soc Nephrol 15:1475–1487. 10.1097/01.asn.0000127988.42710.a7 15153558 10.1097/01.asn.0000127988.42710.a7

[5] Henger A, Huber T, Fischer KG, Nitschke R, Mundel P, Schollmeyer P, Greger R, Pavenstädt H (1997) Angiotensin II increases the cytosolic calcium activity in rat podocytes in culture. Kidney Int 52:687–693. 10.1038/ki.1997.383 9291188 10.1038/ki.1997.383

[6] Binz-Lotter J, Jüngst C, Rinschen MM, Koehler S, Zentis P, Schauss A, Schermer B, Benzing T, Hackl MJ (2020) Injured podocytes are sensitized to angiotensin II–induced calcium signaling. J Am Soc Nephrol 31:532–542. 10.1681/ASN.2019020109 31924670 10.1681/ASN.2019020109 PMC7062224

[7] Bootman MD (2012) Calcium signaling. Cold Spring Harb Perspect Biol 4:a011171. 10.1101/cshperspect.a011171 10.1101/cshperspect.a011171 PMC3385957 22751152

[8] Kerjaschki D (1978) Polycation-induced dislocation of slit diaphragms and formation of cell junctions in rat kidney glomeruli: the effects of low temperature, divalent cations, colchicine, and cytochalasin B. Lab Invest 39:430–440. http://www.ncbi.nlm.nih.gov/pubmed/104090 104090

[9] Winn MP, Conlon PJ, Lynn KL, Farrington MK, Creazzo T, Hawkins AF, Daskalakis N, Kwan SY, Ebersviller S, Burchette JL, Pericak-Vance MA, Howell DN, Vance JM, Rosenberg PB (2005) A mutation in the TRPC6 cation channel causes familial focal segmental glomerulosclerosis. Science 308:1801–1804. 10.1126/science.1106215 15879175 10.1126/science.1106215

[10] Reiser J, Pixley FJ, Hug A, Kriz W, Smoyer WE, Stanley ER, Mundel P (2000) Regulation of mouse podocyte process dynamics by protein tyrosine phosphatases rapid communication. Kidney Int 57:2035–2042. 10.1046/j.1523-1755.2000.00070.x 10792622 10.1046/j.1523-1755.2000.00070.x

[11] Cybulsky AV, Bonventre JV, Quigg RJ, Lieberthal W, Salant DJ (1990) Cytosolic calcium and protein kinase C reduce complement-mediated glomerular epithelial injury. Kidney Int 38:803–811. 10.1038/ki.1990.274 2266662 10.1038/ki.1990.274

[12] Riedl J, Crevenna AH, Kessenbrock K, Yu JH, Neukirchen D, Bista M, Bradke F, Jenne D, Holak TA, Werb Z, Sixt M, Wedlich-Soldner R (2008) Lifeact: a versatile marker to visualize F-actin. Nat Methods 5:605–607. 10.1038/nmeth.1220 18536722 10.1038/nmeth.1220 PMC2814344

[13] Garg P (2018) A review of podocyte biology. Am J Nephrol 47:3–13. 10.1159/000481633 29852492 10.1159/000481633

[14] Burford JL, Villanueva K, Lam L, Riquier-Brison A, Hackl MJ, Pippin J, Shankland SJ, Peti-Peterdi J (2014) Intravital imaging of podocyte calcium in glomerular injury and disease. J Clin Invest 124:2050–2058. 10.1172/JCI71702 24713653 10.1172/JCI71702 PMC4001540

[15] Staruschenko A, Spires D, Palygin O (2019) Role of TRPC6 in progression of diabetic kidney disease. Curr Hypertens Rep 21:48. 10.1007/s11906-019-0960-9 31115705 10.1007/s11906-019-0960-9 PMC6814005

[16] Ilatovskaya DV, Staruschenko A (2015) TRPC6 channel as an emerging determinant of the podocyte injury susceptibility in kidney diseases. Am J Physiol Ren Physiol 309:F393–F397. 10.1152/ajprenal.00186.2015 10.1152/ajprenal.00186.2015 PMC4556891 26084930

[17] Hall G, Wang L, Spurney RF (2019) TRPC channels in proteinuric kidney diseases. Cells 9:44. 10.3390/cells9010044 31877991 10.3390/cells9010044 PMC7016871

[18] Dryer SE, Roshanravan H, Kim EY (2019) TRPC channels: regulation, dysregulation and contributions to chronic kidney disease. Biochim Biophys Acta Mol Basis Dis 1865:1041–1066. 10.1016/j.bbadis.2019.04.001 30953689 10.1016/j.bbadis.2019.04.001

[19] Pablo JL, Greka A (2019) Charting a TRP to novel therapeutic destinations for kidney diseases. Trends Pharmacol Sci 40:911–918. 10.1016/j.tips.2019.10.001 31704171 10.1016/j.tips.2019.10.001 PMC6884692

[20] Daehn IS, Duffield JS (2021) The glomerular filtration barrier: a structural target for novel kidney therapies. Nat Rev Drug Discov 20:770–788. 10.1038/s41573-021-00242-0 34262140 10.1038/s41573-021-00242-0 PMC8278373

[21] Cayouette S, Lussier MP, Mathieu EL, Bousquet SM, Boulay G (2004) Exocytotic insertion of TRPC6 channel into the plasma membrane upon G q protein-coupled receptor activation. J Biol Chem 279:7241–7246. 10.1074/jbc.M312042200 14662757 10.1074/jbc.M312042200

[22] Chaudhuri P, Rosenbaum MA, Sinharoy P, Damron DS, Birnbaumer L, Graham LM (2016) Membrane translocation of TRPC6 channels and endothelial migration are regulated by calmodulin and PI3 kinase activation. Proc Natl Acad Sci U S A 113:2110–2115. 10.1073/pnas.1600371113 26858457 10.1073/pnas.1600371113 PMC4776520

[23] Dietrich A, Mederos Y, Schnitzler M, Emmel J, Kalwa H, Hofmann T, Gudermann T (2003) N-linked protein glycosylation is a major determinant for basal TRPC3 and TRPC6 channel activity. J Biol Chem 278:47842–47852. 10.1074/jbc.M302983200 12970363 10.1074/jbc.M302983200

[24] Hofmann T, Obukhov AG, Schaefer M, Harteneck C, Gudermann T, Schultz G (1999) Direct activation of human TRPC6 and TRPC3 channels by diacylglycerol. Nature 397:259–263. 10.1038/16711 9930701 10.1038/16711

[25] Okada T, Inoue R, Yamazaki K, Maeda A, Kurosaki T, Yamakuni T, Tanaka I, Shimizu S, Ikenaka K, Imoto K, Mori Y (1999) Molecular and functional characterization of a novel mouse transient receptor potential protein homologue TRP7. Ca(2+)-permeable cation channel that is constitutively activated and enhanced by stimulation of G protein-coupled receptor. J Biol Chem 274:27359–27370. 10.1074/jbc.274.39.27359 10488066 10.1074/jbc.274.39.27359

[26] Balla T (2009) Green light to illuminate signal transduction events. Trends Cell Biol 19:575–586. 10.1016/j.tcb.2009.08.001 19818623 10.1016/j.tcb.2009.08.001 PMC2783671

[27] Cheng KT, Ong HL, Liu X, Ambudkar IS (2013) Contribution and regulation of TRPC channels in store-operated Ca2+ entry. Curr Top Membr 71:149–179. 10.1016/B978-0-12-407870-3.00007-X 23890115 10.1016/B978-0-12-407870-3.00007-X PMC3824975

[28] Sonneveld R, Van Der Vlag J, Baltissen MPA, Verkaart SAJ, Wetzels JFM, Berden JHM, Hoenderop JGJ, Nijenhuis T (2014) Glucose specifically regulates TRPC6 expression in the podocyte in an AngII-dependent manner. Am J Pathol 184:1715–1726. 10.1016/j.ajpath.2014.02.008 24731445 10.1016/j.ajpath.2014.02.008

[29] Eckel J, Lavin PJ, Finch EA, Mukerji N, Burch J, Gbadegesin R, Wu G, Bowling B, Byrd A, Hall G, Sparks M, Zhang ZS, Homstad A, Barisoni L, Birbaumer L et al (2011) TRPC6 enhances angiotensin II-induced albuminuria. J Am Soc Nephrol 22:526–535. 10.1681/ASN.2010050522 21258036 10.1681/ASN.2010050522 PMC3060446

[30] Staruschenko A, Ma R, Palygin O, Dryer SE (2023) Ion channels and channelopathies in glomeruli. Physiol Rev 103:787–854. 10.1152/physrev.00013.2022 36007181 10.1152/physrev.00013.2022 PMC9662803

[31] Zhou Y, Castonguay P, Sidhom E-H, Clark AR, Dvela-Levitt M, Kim S, Sieber J, Wieder N, Jung JY, Andreeva S, Reichardt J, Dubois F, Hoffmann SC, Basgen JM, Montesinos MS et al (2017) A small-molecule inhibitor of TRPC5 ion channels suppresses progressive kidney disease in animal models. Science 358:1332–1336. 10.1126/science.aal4178 29217578 10.1126/science.aal4178 PMC6014699

[32] Tao J, Lan Z, Wang Y, Hei H, Tian L, Pan W, Zhang X, Peng W (2016) Large-conductance calcium-activated potassium channels in glomerulus: From cell signal integration to disease. Front Physiol 7:248. 10.3389/fphys.2016.00248 27445840 10.3389/fphys.2016.00248 PMC4915313

[33] Wu P, Wang Y, Davis ME, Zuckerman JE, Chaudhari S, Begg M, Ma R (2015) Store-operated Ca2+ channels in mesangial cells inhibit matrix protein expression. J Am Soc Nephrol 26:2691–2702. 10.1681/ASN.2014090853 25788524 10.1681/ASN.2014090853 PMC4625675

[34] Palygin O, Klemens CA, Isaeva E, Levchenko V, Spires DR, Dissanayake LV, Nikolaienko O, Ilatovskaya DV, Staruschenko A (2021) Characterization of purinergic receptor 2 signaling in podocytes from diabetic kidneys. IScience 24:102528. 10.1016/j.isci.2021.102528 34142040 10.1016/j.isci.2021.102528 PMC8188476

[35] Roshanravan H, Kim EY, Dryer SE (2016) NMDA receptors as potential therapeutic targets in diabetic nephropathy: increased renal NMDA receptor subunit expression in akita mice and reduced nephropathy following sustained treatment with memantine or MK-801. Diabetes 65:3139–3150. 10.2337/db16-0209 27388219 10.2337/db16-0209 PMC5033270

[36] Djenoune L, Tomar R, Dorison A, Ghobrial I, Schenk H, Hegermann J, Beverly-Staggs L, Hidalgo-Gonzalez A, Little MH, Drummond IA (2021) Autonomous calcium signaling in human and zebrafish podocytes controls kidney filtration barrier morphogenesis. J Am Soc Nephrol 32:1697–1712. 10.1681/ASN.2020101525 33911000 10.1681/ASN.2020101525 PMC8425667

[37] Tu YC, Shu HP, Sun LL, Liao QQ, Feng L, Ren M, Yao LJ (2023) The physiopathologic roles of calcium signaling in podocytes. Front Biosci (Landmark Ed) 28:240. 10.31083/j.fbl2810240 37919067 10.31083/j.fbl2810240

[38] Brinkkoetter PT, Ising C, Benzing T (2013) The role of the podocyte in albumin filtration. Nat Rev Nephrol 9:328–336. 10.1038/nrneph.2013.78 23609563 10.1038/nrneph.2013.78

[39] Etienne-Manneville S, Hall A (2002) Rho GTPases in cell biology. Nature 420:629–635. 10.1038/nature01148 12478284 10.1038/nature01148

[40] Raftopoulou M, Hall A (2004) Cell migration: Rho GTPases lead the way. Dev Biol 265:23–32. 10.1016/j.ydbio.2003.06.003 14697350 10.1016/j.ydbio.2003.06.003

[41] Jaffe AB, Hall A (2005) Rho GTPases: Biochemistry and biology. Annu Rev Cell Dev Biol 21:247–269. 10.1146/annurev.cellbio.21.020604.150721 16212495 10.1146/annurev.cellbio.21.020604.150721

[42] Jaffe AB, Hall A (2003) Smurfing at the Leading Edge. Science 302:1690–1691. 10.1126/science.1092874 14657480 10.1126/science.1092874

[43] Ito K, Ger Y, Kawamura S (1986) Actin filament alterations in glomerular epithelial cells of adriamycin-induced nephrotic rats. Acta Pathol Jpn 36:253–260. 10.1111/j.1440-1827.1986.tb01477.x 3518337 10.1111/j.1440-1827.1986.tb01477.x

[44] Whiteside CI, Cameron R, Munk S, Levy J (1993) Podocytic cytoskeletal disaggregation and basement-membrane detachment in puromycin aminonucleoside nephrosis. Am J Pathol 142:1641–1653. http://www.ncbi.nlm.nih.gov/pubmed/8494056 PMC1886918 8494056

[45] Lachapelle M, Bendayan M (1991) Contractile proteins in podocytes: immunocytochemical localization of actin and alpha-actinin in normal and nephrotic rat kidneys. Virchows Arch B Cell Pathol Incl Mol Pathol 60:105–111. 10.1007/BF02899534 1675506 10.1007/BF02899534

[46] Schwarz K, Simons M, Reiser J, Saleem MA, Faul C, Kriz W, Shaw AS, Holzman LB, Mundel P (2001) Podocin, a raft-associated component of the glomerular slit diaphragm, interacts with CD2AP and nephrin. J Clin Invest 108:1621–1629. 10.1172/JCI200112849 11733557 10.1172/JCI12849 PMC200981

[47] Shih NY, Li J, Cotran R, Mundel P, Miner JH, Shaw AS (2001) CD2AP localizes to the slit diaphragm and binds to nephrin via a novel C-terminal domain. Am J Pathol 159:2303–2308. 10.1016/S0002-9440(10)63080-5 11733379 10.1016/S0002-9440(10)63080-5 PMC1850607

[48] Welsch T, Endlich N, Kriz W, Endlich K (2001) CD2AP and p130Cas localize to different F-actin structures in podocytes. Am J Physiol Renal Physiol 281:F769–F777. 10.1152/ajprenal.2001.281.4.F769 11553524 10.1152/ajprenal.2001.281.4.F769

[49] Yuan H, Takeuchi E, Salant DJ (2002) Podocyte slit-diaphragm protein nephrin is linked to the actin cytoskeleton. Am J Physiol Renal Physiol 282:F585–F591. 10.1152/ajprenal.00290.2001 11880318 10.1152/ajprenal.00290.2001

[50] Kerjaschki D (2001) Caught flat-footed: podocyte damage and the molecular bases of focal glomerulosclerosis. J Clin Invest 108:1583–1587. 10.1172/JCI200114629 11733553 10.1172/JCI14629 PMC201002

[51] Wang H-R, Zhang Y, Ozdamar B, Ogunjimi AA, Alexandrova E, Thomsen GH, Wrana JL (2003) Regulation of cell polarity and protrusion formation by targeting RhoA for degradation. Science 302:1775–1779. 10.1126/science.1090772 14657501 10.1126/science.1090772

[52] Ozdamar B, Bose R, Barrios-Rodiles M, Wang H-R, Zhang Y, Wrana JL (2005) Regulation of the polarity protein Par6 by TGFß receptors controls epithelial cell plasticity. Science 307:1603–1609. 10.1126/science.1105718 15761148 10.1126/science.1105718

[53] Asanuma K, Yanagida-Asanuma E, Faul C, Tomino Y, Kim K, Mundel P (2006) Synaptopodin orchestrates actin organization and cell motility via regulation of RhoA signalling. Nat Cell Biol 8:485–491. 10.1038/ncb1400 16622418 10.1038/ncb1400

[54] Jones N, New LA, Fortino MA, Eremina V, Ruston J, Blasutig IM, Aoudjit L, Zou Y, Liu X, Yu GL, Takano T, Quaggin SE, Pawson T (2009) Nck proteins maintain the adult glomerular filtration barrier. J Am Soc Nephrol 20:1533–1543. 10.1681/ASN.2009010056 19443634 10.1681/ASN.2009010056 PMC2709686

[55] Tossidou I, Teng B, Worthmann K, Müller-Deile J, Jobst-Schwan T, Kardinal C, Schroder P, Bolanos-Palmieri P, Haller H, Willerding J, Drost DM, de Jonge L, Reubold T, Eschenburg S, Johnson RI et al (2019) Tyrosine phosphorylation of cd2ap affects stability of the slit diaphragm complex. J Am Soc Nephrol 30:1220–1237. 10.1681/ASN.2018080860 31235616 10.1681/ASN.2018080860 PMC6622410

[56] Kestilä M, Lenkkeri U, Männikkö M, Lamerdin J, McCready P, Putaala H, Ruotsalainen V, Morita T, Nissinen M, Herva R, Kashtan CE, Peltonen L, Holmberg C, Olsen A, Tryggvason K (1998) Positionally cloned gene for a novel glomerular protein–nephrin–is mutated in congenital nephrotic syndrome. Mol Cell 1:575–582. 10.1016/s1097-2765(00)80057-x 9660941 10.1016/s1097-2765(00)80057-x

[57] Krendel M, Leh S, Garone ME, Edwards-Richards A, Lin JJ, Brackman D, Knappskog P, Mikhailov A (2023) Focal segmental glomerulosclerosis and proteinuria associated with Myo1E mutations: novel variants and histological phenotype analysis. Pediatr Nephrol 38:439–449. 10.1007/s00467-022-05634-x 35723736 10.1007/s00467-022-05634-x PMC10506584

[58] Sadowski CE, Lovric S, Ashraf S, Pabst WL, Gee HY, Kohl S, Engelmann S, Vega-Warner V, Fang H, Halbritter J, Somers MJ, Tan W, Shril S, Fessi I, Lifton RP et al (2015) A single-gene cause in 29.5% of cases of steroid-resistant nephrotic syndrome. J Am Soc Nephrol 26:1279–1289. 10.1681/ASN.2014050489 25349199 10.1681/ASN.2014050489 PMC4446877

[59] Blaine J, Dylewski J (2020) Regulation of the actin cytoskeleton in podocytes. Cells 9:1700. 10.3390/cells9071700 10.3390/cells9071700 PMC7408282 32708597

[60] Aramburu J, Heitman J, Crabtree GR (2004) Calcineurin: a central controller of signalling in eukaryotes. Workshop on the calcium/calcineurin/NFAT pathway: Regulation and function. EMBO Rep 5:343–348. 10.1038/sj.embor.7400133 15060569 10.1038/sj.embor.7400133 PMC1299038

[61] Rusnak F, Mertz P (2000) Calcineurin: form and function. Physiol Rev 80:1483–1521. 10.1152/physrev.2000.80.4.1483 11015619 10.1152/physrev.2000.80.4.1483

[62] Wakamatsu A, Fukusumi Y, Hasegawa E, Tomita M, Watanabe T, Narita I, Kawachi H (2016) Role of calcineurin (CN) in kidney glomerular podocyte: CN inhibitor ameliorated proteinuria by inhibiting the redistribution of CN at the slit diaphragm. Physiol Rep 4:e12679. 10.14814/phy2.12679 10.14814/phy2.12679 PMC4814882 27009276

[63] Wang Y, Jarad G, Tripathi P, Pan M, Cunningham J, Martin DR, Liapis H, Miner JH, Chen F (2010) Activation of NFAT signaling in podocytes causes glomerulosclerosis. J Am Soc Nephrol 21:1657–1666. 10.1681/ASN.2009121253 20651158 10.1681/ASN.2009121253 PMC3013542

[64] Clipstone NA, Crabtree GR (1992) Identification of calcineurin as a key signalling enzyme in T-lymphocyte activation. Nature 357:695–697. 10.1038/357695a0 1377362 10.1038/357695a0

[65] Faul C, Donnelly M, Merscher-Gomez S, Chang YH, Franz S, Delfgaauw J, Chang JM, Choi HY, Campbell KN, Kim K, Reiser J, Mundel P (2008) The actin cytoskeleton of kidney podocytes is a direct target of the antiproteinuric effect of cyclosporine A. Nat Med 14:931–938. 10.1038/nm.1857 18724379 10.1038/nm.1857 PMC4109287

[66] Büscher AK, Kranz B, Büscher R, Hildebrandt F, Dworniczak B, Pennekamp P, Kuwertz-Bröking E, Wingen AM, John U, Kemper M, Monnens L, Hoyer PF, Weber S, Konrad M (2010) Immunosuppression and renal outcome in congenital and pediatric steroid-resistant nephrotic syndrome. Clin J Am Soc Nephrol 5:2075–2084. 10.2215/CJN.01190210 20798252 10.2215/CJN.01190210 PMC3001773

[67] Gigante M, Caridi G, Montemurno E, Soccio M, D’Apolito M, Cerullo G, Aucella F, Schirinzi A, Emma F, Massella L, Messina G, de Palo T, Ranieri E, Ghiggeri GM, Gesualdo L (2011) TRPC6 Mutations in children with steroid-resistant nephrotic syndrome and atypical phenotype. Clin J Am Soc Nephrol 6:1626–1634. 10.2215/CJN.07830910 21734084 10.2215/CJN.07830910

[68] Riehle M, Büscher AK, Gohlke BO, Kaßmann M, Kolatsi-Joannou M, Bräsen JH, Nagel M, Becker JU, Winyard P, Hoyer PF, Preissner R, Krautwurst D, Gollasch M, Weber S, Harteneck C (2016) TRPC6 G757D loss-of-function mutation associates with FSGS. J Am Soc Nephrol 27:2771–2783. 10.1681/ASN.2015030318 26892346 10.1681/ASN.2015030318 PMC5004639

[69] Tian D, Jacobo SMP, Billing D, Rozkalne A, Gage SD, Anagnostou T, Pavenstädt H, Hsu H-H, Schlondorff J, Ramos A, Greka A (2010) Antagonistic regulation of actin dynamics and cell motility by TRPC5 and TRPC6 channels. Sci Signal 3:ra77. 10.1126/scisignal.2001200 20978238 10.1126/scisignal.2001200 PMC3071756

[70] Greka A, Mundel P (2012) Calcium regulates podocyte actin dynamics. Semin Nephrol 32:319–326. 10.1016/j.semnephrol.2012.06.003 22958486 10.1016/j.semnephrol.2012.06.003 PMC3581337

[71] Maeda K, Otomo K, Yoshida N, Abu-Asab MS, Ichinose K, Nishino T, Kono M, Ferretti A, Bhargava R, Maruyama S, Bickerton S, Fahmy TM, Tsokos MG, Tsokos GC (2018) CaMK4 compromises podocyte function in autoimmune and nonautoimmune kidney disease. J Clin Invest 128:3445–3459. 10.1172/JCI99507 29985166 10.1172/JCI99507 PMC6063476

[72] Li Q, Gulati A, Lemaire M, Nottoli T, Bale A, Tufro A (2021) Rho-GTPase Activating Protein myosin MYO9A identified as a novel candidate gene for monogenic focal segmental glomerulosclerosis. Kidney Int 99:1102–1117. 10.1016/j.kint.2020.12.022 33412162 10.1016/j.kint.2020.12.022 PMC8076076

[73] Polat OK, Uno M, Maruyama T, Tran HN, Imamura K, Wong CF, Sakaguchi R, Ariyoshi M, Itsuki K, Ichikawa J, Morii T, Shirakawa M, Inoue R, Asanuma K, Reiser J et al (2019) Contribution of coiled-coil assembly to ca2+/ calmodulin-dependent inactivation of TRPC6 channel and its impacts on FSGS-associated phenotypes. J Am Soc Nephrol 30:1587–1603. 10.1681/ASN.2018070756 31266820 10.1681/ASN.2018070756 PMC6727271

[74] Guilluy C, Brégeon J, Toumaniantz G, Rolli-Derkinderen M, Retailleau K, Loufrani L, Henrion D, Scalbert E, Bril A, Torres RM, Offermanns S, Pacaud P, Loirand G (2010) The Rho exchange factor Arhgef1 mediates the effects of angiotensin II on vascular tone and blood pressure. Nat Med 16:183–190. 10.1038/nm.2079 20098430 10.1038/nm.2079

[75] Ohta Y, Hartwig JH, Stossel TP (2006) FilGAP, a Rho- and ROCK-regulated GAP for Rac binds filamin A to control actin remodelling. Nat Cell Biol 8:803–814. 10.1038/ncb1437 16862148 10.1038/ncb1437

[76] Akilesh S, Suleiman H, Yu H, Stander MC, Lavin P, Gbadegesin R, Antignac C, Pollak M, Kopp JB, Winn MP, Shaw AS (2011) Arhgap24 inactivates Rac1 in mouse podocytes, and a mutant form is associated with familial focal segmental glomerulosclerosis. J Clin Invest 121:4127–4137. 10.1172/JCI46458 21911940 10.1172/JCI46458 PMC3195463

[77] Saleem MA, Welsh GI (2019) Podocyte Rho GTPases: new therapeutic targets for nephrotic syndrome?. F1000Res 8:F1000 Faculty Rev-1847. 10.12688/f1000research.20105.1 10.12688/f1000research.20105.1 PMC6833986 31723415

[78] Rovin BH, Adler SG, Barratt J, Bridoux F, Burdge KA, Chan TM, Cook HT, Fervenza FC, Gibson KL, Glassock RJ, Jayne DRW, Jha V, Liew A, Liu ZH, Mejía-Vilet JM et al (2021) KDIGO 2021 clinical practice guideline for the management of glomerular diseases. Kidney Int 100:S1–S276. 10.1016/j.kint.2021.05.021 34556256 10.1016/j.kint.2021.05.021

[79] Ponticelli C, Locatelli F (2018) Glucocorticoids in the treatment of glomerular diseases: pitfalls and pearls. Clin J Am Soc Nephrol 13:815–822. 10.2215/CJN.12991117 29475991 10.2215/CJN.12991117 PMC5969489

[80] Panettieri RA, Schaafsma D, Amrani Y, Koziol-White C, Ostrom R, Tliba O (2019) Non-genomic effects of glucocorticoids: an updated view. Trends Pharmacol Sci 40:38–49. 10.1016/j.tips.2018.11.002 30497693 10.1016/j.tips.2018.11.002 PMC7106476

[81] Guess A, Agrawal S, Wei C-C, Ransom RF, Benndorf R, Smoyer WE (2010) Dose-and time-dependent glucocorticoid receptor signaling in podocytes. Am J Physiol Ren Physiol 299:845–853. 10.1152/ajprenal.00161.2010.-Glu 10.1152/ajprenal.00161.2010 PMC2957255 20630936

[82] Wada T, Pippin JW, Nangaku M, Shankland SJ (2008) Dexamethasone’s prosurvival benefits in podocytes require extracellular signal-regulated kinase phosphorylation. Nephron Exp Nephrol 109:e8–e19. 10.1159/000131892 18480613 10.1159/000131892

[83] Xing CY, Saleem MA, Coward RJ, Ni L, Witherden IR, Mathieson PW (2006) Direct effects of dexamethasone on human podocytes. Kidney Int 70:1038–1045. 10.1038/sj.ki.5001655 16837924 10.1038/sj.ki.5001655

[84] van den Broek M, Smeets B, Schreuder MF, Jansen J (2022) The podocyte as a direct target of glucocorticoids in nephrotic syndrome. Nephrol Dial Transplant 37:1808–1815. 10.1093/ndt/gfab016 33515261 10.1093/ndt/gfab016

[85] Yu S, Yu L (2012) Dexamethasone resisted podocyte injury via stabilizing TRPC6 expression and distribution. Evid Based Complement Altern Med 2012:652059. 10.1155/2012/652059 10.1155/2012/652059 PMC3321534 22545060

[86] Castellino F, Heuser J, Marchetti S, Bruno B, Luini A (1992) Glucocorticoid stabilization of actin filaments: a possible mechanism for inhibition of corticotropin release. Proc Natl Acad Sci U S A 89:3775–3779. 10.1073/pnas.89.9.3775 1315038 10.1073/pnas.89.9.3775 PMC525573

[87] Koukouritaki SB, Lianos EA (1999) Glucocorticoid effect on human mesangial cell cytoskeletal proteins. J Lab Clin Med 133:378–383. 10.1016/s0022-2143(99)90069-0 10218769 10.1016/s0022-2143(99)90069-0

[88] Yamauchi K, Takano Y, Kasai A, Hayakawa K, Hiramatsu N, Enomoto N, Yao J, Kitamura M (2006) Screening and identification of substances that regulate nephrin gene expression using engineered reporter podocytes. Kidney Int 70:892–900. 10.1038/sj.ki.5001625 16820792 10.1038/sj.ki.5001625

[89] Ohashi T, Uchida K, Uchida S, Sasaki S, Nitta K (2011) Dexamethasone increases the phosphorylation of nephrin in cultured podocytes. Clin Exp Nephrol 15:688–693. 10.1007/s10157-011-0479-0 21695412 10.1007/s10157-011-0479-0

[90] Yu-Shengyou LY (2013) Dexamethasone inhibits podocyte apoptosis by stabilizing the PI3K/Akt signal pathway. Biomed Res Int 2013:326986. 10.1155/2013/326986 23710442 10.1155/2013/326986 PMC3655450

[91] Bohr DC, Koch M, Kritzenberger M, Fuchshofer R, Tamm ER (2011) Increased expression of olfactomedin-1 and myocilin in podocytes during puromycin aminonucleoside nephrosis. Nephrol Dial Transplant 26:83–92. 10.1093/ndt/gfq366 20595200 10.1093/ndt/gfq366

[92] Mallipattu SK, Guo Y, Revelo MP, Roa-Peña L, Miller T, Ling J, Shankland SJ, Bialkowska AB, Ly V, Estrada C, Jain MK, Lu Y, Ma’Ayan A, Mehrotra A, Yacoub R et al (2017) Krüppel-like factor 15 mediates glucocorticoid-induced restoration of podocyte differentiation markers. J Am Soc Nephrol 28:166–184. 10.1681/ASN.2015060672 27288011 10.1681/ASN.2015060672 PMC5198263

[93] Mallipattu SK, Liu R, Zheng F, Narla G, Ma’ayan A, Dikman S, Jain MK, Saleem M, D’Agati V, Klotman P, Chuang PY, He JC (2012) Krüppel-like factor 15 (KLF15) is a key regulator of podocyte differentiation. J Biol Chem 287:19122–19135. 10.1074/jbc.M112.345983 22493483 10.1074/jbc.M112.345983 PMC3365945

[94] Han SS, Yu MY, Yoo KD, Lee JP, Kim DK, Kim YS, Yang SH (2018) Loss of KLF15 accelerates chronic podocyte injury. Int J Mol Med 42:1593–1602. 10.3892/ijmm.2018.3726 29901095 10.3892/ijmm.2018.3726

[95] Liu H, Gao X, Xu H, Feng C, Kuang X, Li Z, Zha X (2012) α-Actinin-4 is involved in the process by which dexamethasone protects actin cytoskeleton stabilization from adriamycin-induced podocyte injury. Nephrology 17:669–675. 10.1111/j.1440-1797.2012.01645.x 22804863 10.1111/j.1440-1797.2012.01645.x

[96] McCaffrey JC, Webb NJ, Poolman TM, Fresquet M, Moxey C, Zeef LAH, Donaldson IJ, Ray DW, Lennon R (2017) Glucocorticoid therapy regulates podocyte motility by inhibition of Rac1. Sci Rep 7:6725. 10.1038/s41598-017-06810-y 28751734 10.1038/s41598-017-06810-y PMC5532274

[97] Lewko B, Waszkiewicz A, Maryn A, Gołos M, Latawiec E, Daca A, Witkowski JM, Angielski S, Stępiński J (2015) Dexamethasone-dependent modulation of cyclic GMP synthesis in podocytes. Mol Cell Biochem 409:243–253. 10.1007/s11010-015-2528-6 26272337 10.1007/s11010-015-2528-6 PMC4589550

[98] Ransom RF, Lam NG, Hallett MA, Atkinson SJ, Smoyer WE (2005) Glucocorticoids protect and enhance recovery of cultured murine podocytes via actin filament stabilization. Kidney Int 68:2473–2483. 10.1111/j.1523-1755.2005.00723.x 16316324 10.1111/j.1523-1755.2005.00723.x

[99] Agrawal S, Chanley MA, Westbrook D, Nie X, Kitao T, Guess AJ, Benndorf R, Hidalgo G, Smoyer WE (2016) Pioglitazone enhances the beneficial effects of glucocorticoids in experimental nephrotic syndrome. Sci Rep 6:24392. 10.1038/srep24392 27142691 10.1038/srep24392 PMC4855145

[100] Vassiliadis J, Bracken C, Matthews D, O’Brien S, Schiavi S, Wawersik S (2011) Calcium mediates glomerular filtration through calcineurin and mTORC2/Akt signaling. J Am Soc Nephrol 22:1453–1461. 10.1681/ASN.2010080878 21784900 10.1681/ASN.2010080878 PMC3148700

[101] Shen X, Jiang H, Ying M, Xie Z, Li X, Wang H, Zhao J, Lin C, Wang Y, Feng S, Shen J, Weng C, Lin W, Wang H, Zhou Q et al (2016) Calcineurin inhibitors cyclosporin A and tacrolimus protect against podocyte injury induced by puromycin aminonucleoside in rodent models. Sci Rep 6:32087. 10.1038/srep32087 27580845 10.1038/srep32087 PMC5007516

[102] Nijenhuis T, Sloan AJ, Hoenderop JGJ, Flesche J, Van Goor H, Kistler AD, Bakker M, Bindels RJM, De Boer RA, Möller CC, Hamming I, Navis G, Wetzels JFM, Berden JHM, Reiser J et al (2011) Angiotensin II contributes to podocyte injury by increasing TRPC6 expression via an NFAT-mediated positive feedback signaling pathway. Am J Pathol 179:1719–1732. 10.1016/j.ajpath.2011.06.033 21839714 10.1016/j.ajpath.2011.06.033 PMC3181349

[103] D’Agati VD, Kaskel FJ, Falk RJ (2011) Focal segmental glomerulosclerosis. N Engl J Med 365:2398–2411. 10.1056/NEJMra1106556 22187987 10.1056/NEJMra1106556

[104] Yasuda H, Fukusumi Y, Ivanov V, Zhang Y, Kawachi H (2021) Tacrolimus ameliorates podocyte injury by restoring FK506 binding protein 12 (FKBP12) at actin cytoskeleton. FASEB J 35:e21983. 10.1096/fj.202101052R 34662453 10.1096/fj.202101052R

[105] Liao R, Liu Q, Zheng Z, Fan J, Peng W, Kong Q, He H, Yang S, Chen W, Tang X, Yu X (2015) Tacrolimus protects podocytes from injury in lupus nephritis partly by stabilizing the cytoskeleton and inhibiting podocyte apoptosis. PLoS One 10:e0132724. 10.1371/journal.pone.0132724 26161538 10.1371/journal.pone.0132724 PMC4498640

[106] Wu J, Zheng C, Wang X, Yun S, Zhao Y, Liu L, Lu Y, Ye Y, Zhu X, Zhang C, Shi S, Liu Z (2015) MicroRNA-30 family members regulate calcium/calcineurin signaling in podocytes. J Clin Invest 125:4091–4106. 10.1172/JCI81061 26436650 10.1172/JCI81061 PMC4639992

[107] Fu J, Wang Z, Lee K, Wei C, Liu Z, Zhang M, Zhou M, Cai M, Zhang W, Chuang PY, Ma’ayan A, He JC, Liu Z (2018) Transcriptomic analysis uncovers novel synergistic mechanisms in combination therapy for lupus nephritis. Kidney Int 93:416–429. 10.1016/j.kint.2017.08.031 29102373 10.1016/j.kint.2017.08.031

[108] Eugui EM, Mirkovich A, Allison AC (1991) Lymphocyte-selective antiproliferative and immunosuppressive effects of mycophenolic acid in mice. Scand J Immunol 33:175–183. 10.1111/j.1365-3083.1991.tb03747.x 2017655 10.1111/j.1365-3083.1991.tb03747.x

[109] Hackl A, Ehren R, Weber LT (2017) Effect of mycophenolic acid in experimental, nontransplant glomerular diseases: new mechanisms beyond immune cells. Pediatr Nephrol 32:1315–1322. 10.1007/s00467-016-3437-y 27312386 10.1007/s00467-016-3437-y

[110] Abo Zed SED, Hackl A, Bohl K, Ebert L, Kieckhöfer E, Müller C, Becker K, Fink G, Nüsken K-D, Nüsken E, Müller R-U, Schermer B, Weber LT (2023) Mycophenolic acid directly protects podocytes by preserving the actin cytoskeleton and increasing cell survival. Sci Rep 13:4281. 10.1038/s41598-023-31326-z 36922538 10.1038/s41598-023-31326-z PMC10017704

[111] Hackl A, Nüsken E, Voggel J, Abo Zed SED, Binz-Lotter J, Unnersjö-Jess D, Müller C, Fink G, Bohl K, Wiesner E, Diefenhardt P, Dafinger C, Chen H, Wohlfarth M, Müller RU et al (2023) The effect of mycophenolate mofetil on podocytes in nephrotoxic serum nephritis. Sci Rep 13:14167. 10.1038/s41598-023-41222-1 37644089 10.1038/s41598-023-41222-1 PMC10465485

[112] Salama AD, Pusey CD (2006) Drug Insight: rituximab in renal disease and transplantation. Nat Clin Pract Nephrol 2:221–230. 10.1038/ncpneph0133 16932428 10.1038/ncpneph0133

[113] Benz K, Dötsch J, Rascher W, Stachel D (2004) Change of the course of steroid-dependent nephrotic syndrome after rituximab therapy. Pediatr Nephrol 19:794–797. 10.1007/s00467-004-1434-z 15071769 10.1007/s00467-004-1434-z

[114] Fornoni A, Sageshima J, Wei C, Merscher-Gomez S, Aguillon-Prada R, Jauregui AN, Li J, Mattiazzi A, Ciancio G, Chen L, Zilleruelo G, Abitbol C, Chandar J, Seeherunvong W, Ricordi C et al (2011) Rituximab targets podocytes in recurrent focal segmental glomerulosclerosis. Sci Transl Med 3:85ra46. 10.1126/scitranslmed.3002231 21632984 10.1126/scitranslmed.3002231 PMC3719858

[115] Yoo TH, Pedigo CE, Guzman J, Correa-Medina M, Wei C, Villarreal R, Mitrofanova A, Leclercq F, Faul C, Li J, Kretzler M, Nelson RG, Lehto M, Forsblom C, Groop PH et al (2015) Sphingomyelinase-like phosphodiesterase 3b expression levels determine podocyte injury phenotypes in glomerular disease. J Am Soc Nephrol 26:133–147. 10.1681/ASN.2013111213 24925721 10.1681/ASN.2013111213 PMC4279736

[116] Basu B, Angeletti A, Islam B, Ghiggeri GM (2022) New and old anti-CD20 monoclonal antibodies for nephrotic syndrome. Where we are? Front Immunol 13:805697. 10.3389/fimmu.2022.805697 35222385 10.3389/fimmu.2022.805697 PMC8873567

[117] Trautmann A, Schnaidt S, Lipska-Ziȩtkiewicz BS, Bodria M, Ozaltin F, Emma F, Anarat A, Melk A, Azocar M, Oh J, Saeed B, Gheisari A, Caliskan S, Gellermann J, Higuita LMS et al (2017) Long-term outcome of steroid-resistant nephrotic syndrome in children. J Am Soc Nephrol 28:3055–3065. 10.1681/ASN.2016101121 28566477 10.1681/ASN.2016101121 PMC5619960

[118] Büscher AK, Beck BB, Melk A, Hoefele J, Kranz B, Bamborschke D, Baig S, Lange-Sperandio B, Jungraithmayr T, Weber LT, Kemper MJ, Tӧnshoff B, Hoyer PF, Konrad M, Weber S (2016) Rapid response to cyclosporin and favorable renal outcome in nongenetic versus genetic steroid–resistant nephrotic syndrome. Clin J Am Soc Nephrol 11:245–253. 10.2215/CJN.07370715 26668027 10.2215/CJN.07370715 PMC4741047

[119] Gellermann J, Stefanidis CJ, Mitsioni A, Querfeld U (2010) Successful treatment of steroid-resistant nephrotic syndrome associated with WT1 mutations. Pediatr Nephrol 25:1285–1289. 10.1007/s00467-010-1468-3 20191369 10.1007/s00467-010-1468-3

[120] Sinha A, Sharma S, Gulati A, Sharma A, Agarwala S, Hari P, Bagga A (2010) Frasier syndrome: Early gonadoblastoma and cyclosporine responsiveness. Pediatr Nephrol 25:2171–2174. 10.1007/s00467-010-1518-x 20419325 10.1007/s00467-010-1518-x

