# Supplementary material for: The Ca2+-actin-cytoskeleton axis in podocytes is an important, non-immunologic target of immunosuppressive therapy in proteinuric kidney diseases
Source: Pediatr Nephrol. 2025 Jan 25;40(9):2729–39. doi: 10.1007/s00467-025-06670-z (PMC12296978; doi:10.1007/s00467-025-06670-z)
Supplement: Supplementary file 1 — Graphical abstract (PPTX 355 KB) [file 467_2025_6670_MOESM1_ESM.pptx]

## Slide 1
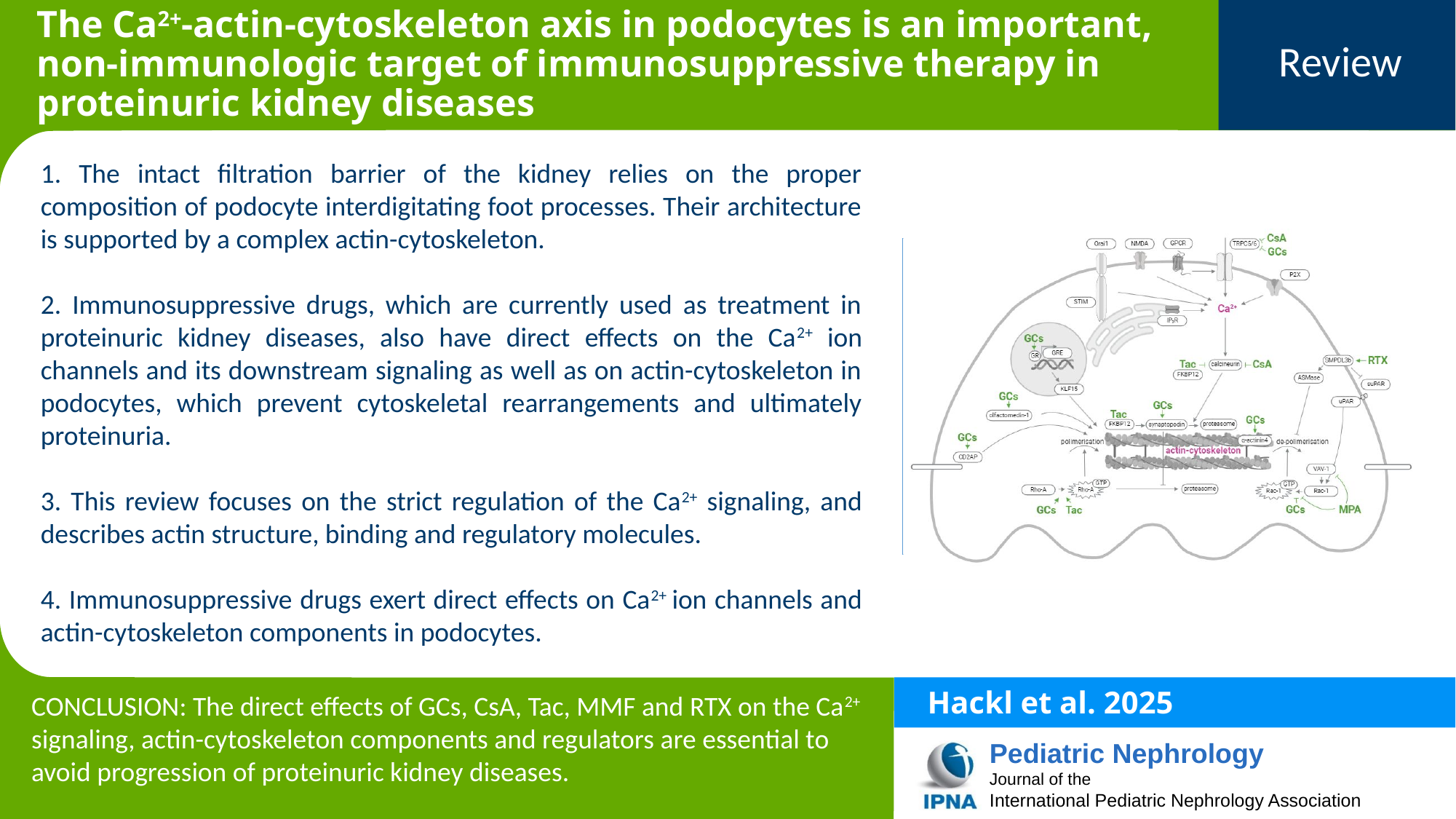

The Ca2+-actin-cytoskeleton axis in podocytes is an important, non-immunologic target of immunosuppressive therapy in proteinuric kidney diseases
1. The intact filtration barrier of the kidney relies on the proper composition of podocyte interdigitating foot processes. Their architecture is supported by a complex actin-cytoskeleton.
2. Immunosuppressive drugs, which are currently used as treatment in proteinuric kidney diseases, also have direct effects on the Ca2+ ion channels and its downstream signaling as well as on actin-cytoskeleton in podocytes, which prevent cytoskeletal rearrangements and ultimately proteinuria.
3. This review focuses on the strict regulation of the Ca2+ signaling, and describes actin structure, binding and regulatory molecules.
4. Immunosuppressive drugs exert direct effects on Ca2+ ion channels and actin-cytoskeleton components in podocytes.
Consider including a representative figure or table from your Review article, if relevant, and if you have the requisite permissions.
Hackl et al. 2025
CONCLUSION: The direct effects of GCs, CsA, Tac, MMF and RTX on the Ca2+ signaling, actin-cytoskeleton components and regulators are essential to avoid progression of proteinuric kidney diseases.
